# Choline and betaine consumption lowers cancer risk: a meta-analysis of epidemiologic studies

**DOI:** 10.1038/srep35547

**Published:** 2016-10-19

**Authors:** Shanwen Sun, Xiao Li, Anjing Ren, Mulong Du, Haina Du, Yongqian Shu, Lingjun Zhu, Wei Wang

**Affiliations:** 1 Department of Oncology, The First Affiliated Hospital of Nanjing Medical University, Nanjing, China; 2 Department of Pathology, The First Affiliated Hospital of Nanjing Medical University, Nanjing, China; 3 Department of Environmental Genomics, Jiangsu Key Laboratory of Cancer Biomarkers, Prevention and Treatment, Cancer Center, Nanjing Medical University, Nanjing, China; 4 Department of Genetic Toxicology, the Key Laboratory of Modern Toxicology of Ministry of Education, School of Public Health, Nanjing Medical University, Nanjing, China; 5 Department of Oncology, Nanjing Hospital of T.C.M, Nanjing, China; 6 Department of Thoracic Surgery, The First Affiliated Hospital of Nanjing Medical University, Nanjing, China

## Abstract

A number of human and animal *in vitro* or *in vivo* studies have investigated the relationship between dietary choline and betaine and cancer risk, suggesting that choline and betaine consumption may be protective for cancer. There are also a few epidemiologic studies exploring this relationship, however, with inconsistent conclusions. The PubMed and Embase were searched, from their inception to March 2016, to identify relevant studies and we brought 11 articles into this meta-analysis eventually. The pooled relative risks (RRs) of cancer for the highest versus the lowest range were 0.82 (95% CI, 0.70 to 0.97) for choline consumption only, 0.86 (95%CI, 0.76 to 0.97) for betaine consumption only and 0.60 (95%CI, 0.40 to 0.90) for choline plus betaine consumption, respectively. Significant protective effect of dietary choline and betaine for cancer was observed when stratified by study design, location, cancer type, publication year, sex and quality score of study. An increment of 100 mg/day of choline plus betaine intake helped reduce cancer incidence by 11% (0.89, 95% CI, 0.87 to 0.92) through a dose-response analysis. To conclude, choline and betaine consumption lowers cancer incidence in this meta-analysis, but further studies are warranted to verify the results.

Cancer is a major cause of death on a global scale. According to the GLOBOCAN estimates, new cancer cases and deaths are approximately 14.1 million and 8.2 million in 2012 worldwide^1^. To slow and even reverse the global trend of increasing in cancer ultimately, preventive measures could provide the merely possible approach^2^.

In the process of cancer prevention, dietary factors have long been regarded as a quite crucial role, among which choline and betaine (choline’s metabolites in the liver and kidney) are likely to be essential and protective nutrients. Choline can be obtained from diet or produced by denovo synthesis in tissues whereas betaine can only be got from diet. Choline has a wide variety of functions, such as maintaining the structural integrity of cells, affecting signaling and transport across membranes and serving as a basic component of the neurotransmitter acetylcholine. Moreover, both choline and betaine participate in one-carbon metabolism, as major methyl-group donors^3^. Evidence from some humans and animals studies has demonstrated that choline insufficiency alters the structure of DNA and histones, resulting in DNA strand breaks^4^,^5^.

So far, there have been a number of epidemiologic studies exploring whether dietary consumption of choline and betaine is associated with the risk of cancer but the results are conflicting. Also, to our knowledge, no article has been found to attempt to make a summary of the results. Hence, it will be of interest to evaluate whether the consumption of choline and betaine is one of the dietary factors that are related with cancer incidence, on the basis of present epidemiologic evidence. To pool the results of relevant reports on the association and evaluate the dose-response relationship between choline and betaine consumption and the risk of cancer as well, we conducted this quantitative meta-analysis.

## Results

### Literature search

**Figure 1:**
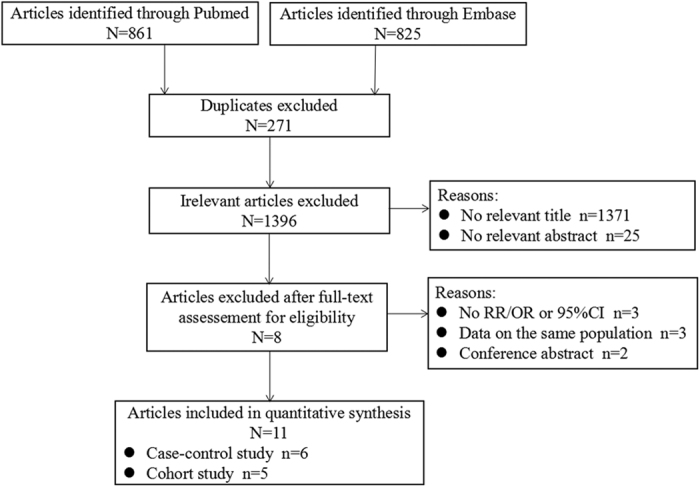
The process diagram of article search and selection in the meta-analysis.

Figure 1 tells the detailed procedures of how we searched and selected relevant articles. In brief, we retrieved 861 articles from Pubmed and 825 articles from Embase, 1,686 articles in total, of which 271 articles were excluded due to duplication. Nineteen articles were left for further evaluation of the full text after screening the titles or abstracts of the remaining articles. We further eliminated 8 articles owing to reasons as listed below: no odds ratio (OR)/relative risk (RR) or 95% confidence interval (CI) reported (n = 3); data on the same population (n = 3); conference abstract lacking enough details for quality assessment (n = 2). Finally, we included 11 articles^6^,^7^,^8^,^9^,^10^,^11^,^12^,^13^,^14^,^15^,^16^ in accordance with the inclusion criteria without additional articles from the reference review. Among these articles, one paper by Ibiebele *et al.*^7^ reported two outcomes of esophageal cancer on the basis of its two subtypes: esophageal adenocarcinoma (EAC) and esophageal squamous cell carcinoma (ESCC); the article by Zhang *et al.*^10^ reported data from a two-stage case-control; another entry by Cho *et al.*^16^ provided information on males and females separately. Thus, our meta-analysis included 14 comparisons.

### Study characteristics and quality assessment

Characteristics extracted from the included 11 articles were displayed in Table 1. Of these articles published between 2007 and 2015, 6 were case-control studies and 5 were cohort studies. In aggregate, we documented 14,488 cases among 513,390 participants, of which the number varied from 738 to 159,957. Seven studies were carried out in the USA, three studies in China and only one study in Australia. The range of the age of all participants was from 18 to 98 years if not considering the article by Ying *et al.*^8^ in which there was no age restrictions. Four studies included both males and females, five studies included only females, one study included only males and one study provided information on males and females separately. In these studies, the role of choline and betaine was investigated in colorectal cancer, breast cancer, esophageal cancer, lung cancer, nasopharyngeal carcinoma, epithelial ovarian cancer and renal cell cancer. To estimate dietary choline and betaine intake, all studies used the Food Frequency Questionnaire (FFQ). Categories of choline and betaine consumption were divided into fourths or fifths. All studies provided adjusted risk estimates.

Supplemental Tables 1 and 2 summarized quality scores of case-control studies and cohort studies, respectively. The quality scores of all studies ranged from 6 to 9. The median score of case-control studies was 6.5 and that of cohort studies was 9. Three case-control studies and all cohort studies were of high quality.

### Choline consumption and cancer risk

**Figure 2:**
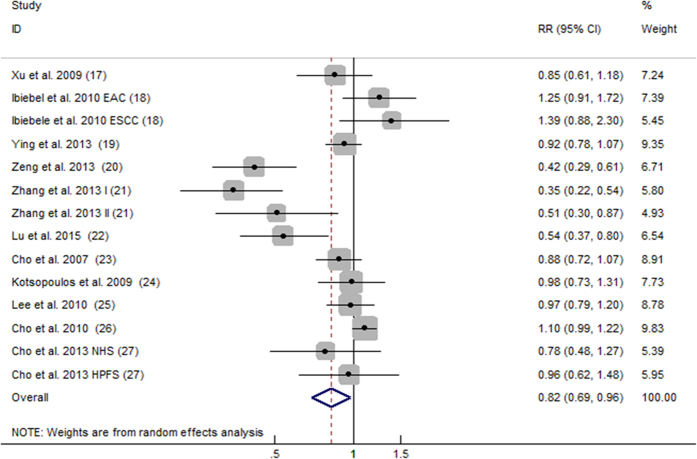
Forest plot of highest versus lowest categories of choline consumption on cancer risk. RR, relative risk; CI, confidence interval; EAC, esophageal adenocarcinoma; ESCC, esophageal squamous cell carcinoma; I, stage one; II stage two; HPFS, The Health Professionals Follow-up Study; NHS, The Nurses’ Health Study.

**Figure 3:**
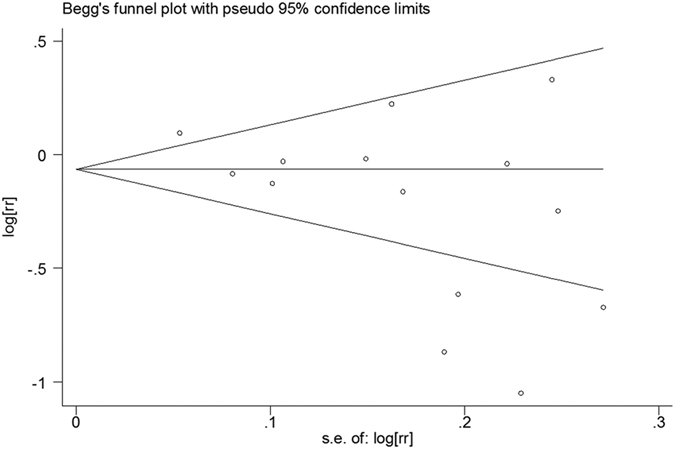
Begg’s funnel plot for publication bias test of the relationship between choline consumption and cancer risk.

All 14 comparisons from 11 articles included reported the relationship between dietary consumption of choline and cancer risk. Overall, for the highest versus the lowest choline consumption, the pooled RR was 0.82 (95%CI, 0.69 to 0.96; Fig. 2) with significant heterogeneity among the 14 comparisons (*P* < 0.001, *I^2^* = 80.2%). According to the Begg’s funnel plot (Fig. 3) and Egger’s test (*P* = 0.030), the publication bias existed.

### Betaine consumption and cancer risk

**Figure 4:**
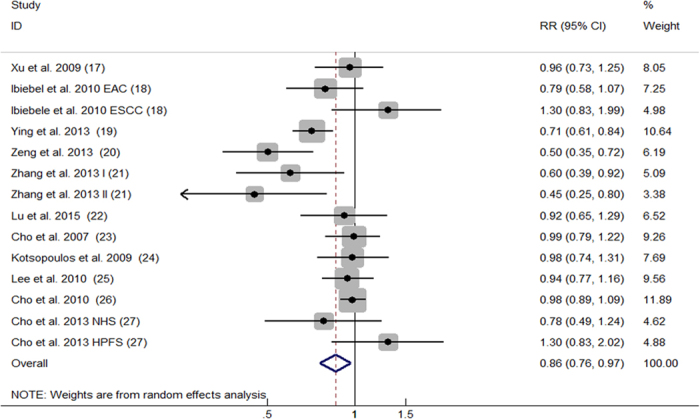
Forest plot of highest versus lowest categories of betaine consumption on cancer risk. RR, relative risk; CI, confidence interval; EAC, esophageal adenocarcinoma; ESCC, esophageal squamous cell carcinoma; I, stage one; II stage two; HPFS, The Health Professionals Follow-up Study; NHS, The Nurses’ Health Study.

**Figure 5:**
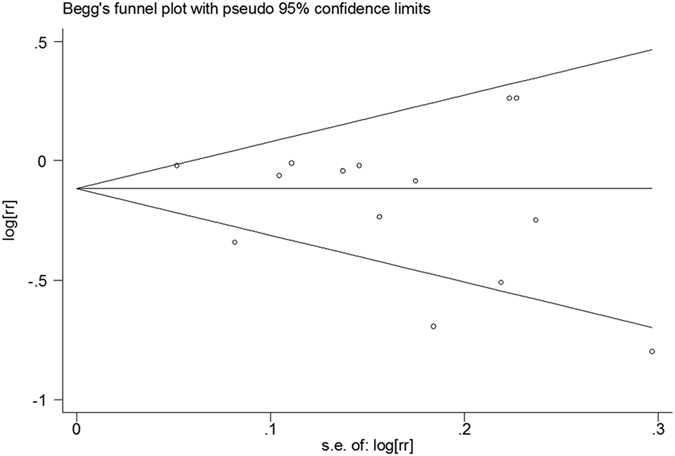
Begg’s funnel plot for publication bias test of the relationship between betaine consumption and cancer risk.

The 11 articles with 14 comparisons also reported the relationship between dietary consumption of betaine and cancer risk. For the highest versus the lowest betaine consumption, the pooled RR was 0.86 (95%CI, 0.76 to 0.97; Fig. 4) with significant heterogeneity among the 14 comparisons (*P* < 0.001, *I^2^* = 65.8%). Additionally, no evidence of publication bias was observed from the Begg’s funnel plot (Fig. 5) and Egger’s test (*P* = 0.319).

### Choline plus betaine consumption and cancer risk

**Figure 6:**
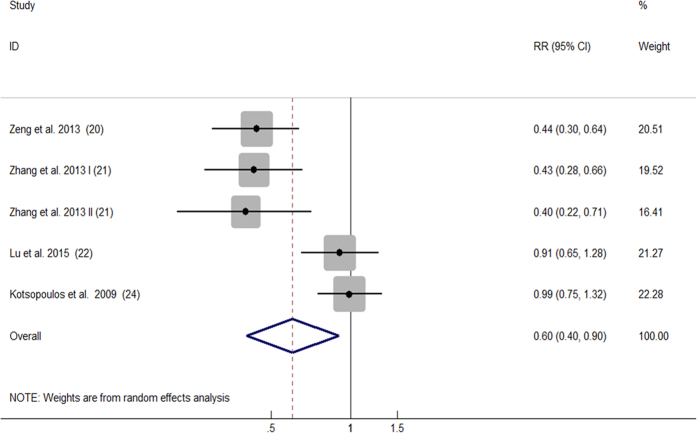
Forest plot of highest versus lowest categories of choline plus betaine consumption on cancer risk. RR, relative risk; CI, confidence interval; I, stage one; II stage two.

Only 5 comparisons from 4 articles^9^,^10^,^11^,^13^ presented results of dietary consumption of choline and betaine combined and cancer risk. For the highest versus the lowest choline plus betaine consumption, the pooled RR was 0.60 (95%CI, 0.40 to 0.90; Fig. 6) with significant heterogeneity among the 5 comparisons (*P* < 0.001, *I^2^* = 82.0%). Publication bias was neither investigated graphically (Begg’s funnel plot) nor by testing (Egger’s test) because the small number of studies limits the usefulness of these methods.

### Dose-response analysis

Only 3 comparisons from 2 case-controls^9^,^10^ were eligible for exploring the dose-response relationship between dietary consumption of choline plus betaine and cancer risk. The summary RR for an increment of 100 mg/day of choline plus betaine consumption was 0.89 (95% CI, 0.87 to 0.92) without heterogeneity (*P* = 0.965, *I^2^* = 0%).

### Stratified analysis

For the purpose of exploring the sources of heterogeneity among the primary results, we conducted stratified analyses for choline consumption only, betaine consumption only as well as choline plus betaine consumption, respectively (Table 2). Significant effect of protection for cancer was revealed in hospital-based case-control studies: 0.52 (95%CI, 0.30 to 0.90) for choline consumption only, 0.60 (95%CI, 0.49 to 0.75) for betaine consumption only and 0.43 (95%CI, 0.33 to 0.55) for choline plus betaine consumption. When stratifying by the location, the RRs were 0.45 (95% CI, 0.36 to 0.55) for choline consumption only, 0.61 (95% CI, 0.44 to 0.85) for betaine consumption only and 0.52 (95% CI, 0.34 to 0.80) for choline plus betaine consumption, all producing significant reduction in cancer risk in China. In the subgroup analysis of cancer type, it was found significantly protective effect for breast cancer only in dietary consumption of choline and betaine combined (RR 0.42, 95%CI, 0.30 to 0.59). The risk for developing cancer was significantly lower in female only (RR 0.78, 95%CI, 0.61 to 0.99) for choline consumption only as well as male and female (RR 0.78, 95%CI, 0.62 to 1.00) for betaine consumption only. Besides, the protective effect for cancer was also observed in these subgroups (publication year after 2010; quality score ≥ 7 stars).

### Meta-regression analysis

As shown in Supplemental Table 3, the meta-regression indicated that location (*P* < 0.001) and publication year (*P* = 0.008) but not the study design were significant sources of heterogeneity in the relationship of choline consumption only and the risk of developing cancer. The estimated between-study variance (**τ^2^**) was decreased from 0.069 to 0.004 (location) and 0.054 (publication year). Moreover, the location alone could explain 94.20% and the publication year could alone explain 21.74% of the **τ^2^**. Hence, for the relationship of the consumption of choline only and cancer risk, the location was the main source of the heterogeneity. As the same time, for the relationship of betaine consumption only and cancer risk, meta-regression indicated that study design (*P* = 0.045), location (*P* = 0.016) and publication year (*P* = 0.006) were all significant sources of heterogeneity. The **τ^2^** was reduced from 0.031 to 0.009 (study design), 0.018 (location) and 0 (publication year). Besides, the study design alone could explain 70.97%, the location alone could explain 41.94% and the publication year could alone explain 100% of the **τ^2^**. Thus, the heterogeneity across the comparisons about the relationship of betaine consumption only and cancer risk was almost entirely from publication year. On account of only 5 comparisons from 4 articles included in the relationship between consumption of choline and betaine combined and cancer risk, we did not run the meta-regression.

### Sensitivity analysis

We also analyzed the sensitivity, attempting to explain the heterogeneity, examine whether varying in the criteria of inclusion had influence over the overall results and confirm the robustness of our results by omitting 1 comparison at every turn and recalculating the pooled relative risks for the remaining. Inclusion of another 3 articles^17^,^18^,^19^ that reported the hazard ratio (HR) with the corresponding 95% CI yielded similar results: a RR of 0.86 (95% CI, 0.74 to 0.99) with substantial evidence of heterogeneity (*P* < 0.001, *I^2^* = 80.0%) for choline consumption only and a RR of 0.87 (95% CI, 0.77 to 0.97) with substantial evidence of heterogeneity (*P* = 0.001, *I^2^* = 61.9%) for betaine consumption only. Under the circumstance of omitting any single comparison, the overall results did not materially alter, with ranges of the pooled RRs from 0.79 (95%CI, 0.67 to 0.94) to 0.87 (95%CI, 0.75 to 1.00) for choline consumption only (Supplemental Figure 1) and from 0.84 (95%CI, 0.74 to 0.95) to 0.89 (95%CI, 0.79 to 1.00) for betaine consumption only (Supplemental Figure 2). For consumption of choline plus betaine (Supplemental Figure 3), the range was from 0.52 (95%CI, 0.34 to 0.80) to 0.65 (95%CI, 0.42 to 1.02) and exclusion of any one of the 3 comparisons from articles by Zeng *et al.*^9^ and Zhang *et al.*^10^ influenced the overall risk estimates. The pooled RRs for the remainders were 0.65 (95%CI, 0.42 to 1.02), 0.65 (95%CI, 0.42 to 1.02) and 0.65 (95%CI, 0.42 to 1.01), respectively, with no heterogeneity changing.

## Discussion

Findings from our meta-analysis manifested that high choline and betaine consumption contributed to cancer prevention. Moreover, the dose-response analysis further demonstrated the protective effect from choline and betaine consumption towards cancer occurrence: an increase in choline plus betaine consumption of 100 mg/day significantly lowered the chance of developing cancer by 11%.

Homocysteine depends on a one-carbon unit to produce methionine and then generate S-adenosylmethionine (SAM), the universal methyl donor, which contributes to methylation actions, such as histone and DNA methylation^20^. Just like 5-methyl tetrahydrofolate, choline can offer the one-carbon unit when oxidized to betaine. Therefore, in case that this pathway of one-carbon metabolism gets disrupted, it will exert an influence over the process of DNA synthesis and repair, as well as the genetic expression regulation by means of methylation, and consequently promotes carcinogenesis. Moreover, choline metabolism and folate metabolism are closely connected, interacting at the point of the conversion from homocysteine to methionine^2^. A few studies on animals and humans have shown that dietary choline and folate are supplementary to each other if either of them gets in a state of deprivation^21^,^22^,^23^. In other words, diets of choline deficiency also result in the deficiency of tissue folate^22^, which plays a vital role in DNA synthesis and one-carbon metabolism when converted into the aforementioned 5-methyl tetrahydrofolate. Furthermore, in animals studies, choline deficiency causes cancer via inducing dysfunction in mitochondria and excessive production of reactive oxygen species (ROS)^24^,^25^, which has long been considered to be one of mechanisms in promoting cancer^26^,^27^. In addition, Ghoshal *et al.* have reported that in experimental animals, a 0.8% supplemented dietary consumption of choline aids in the action of cancer prevention in complete^28^.

The significant protective effect of choline and betaine consumption against cancer was weaker in cohort studies in the subgroup analysis of study design. The discrepant results could be explained by the retrospective nature of case-control studies which were more inclined to have greater recall and selection biases and in which, cancer cases were more likely to change their eating habits and dietary patterns because of information of choline and betaine consumption was gathered in their post-cancer diagnosis life. Besides, we found results from the subgroup analysis of the location which studies conducted in suggest that people settling in China were greatly protected from the risk of developing cancer (a 55% compared with 18% risk reduction in for choline consumption only, a 39% compared with 14% risk reduction for betaine consumption only and a 48% compared with 40% risk reduction for choline plus betaine consumption) and we could not find the analogous results in studies conducted in non-China. These inconsistent findings may have been attributed to differences in the compositions of the diets, diverse eating habits, dissimilar susceptibility to cancer of Chinese and non-Chinese as well as, to some degree, differences stemming from levels of scientific researches in different regions. Additionally, in the separate analysis of cancer type, dietary choline and betaine separately did not show the protective effect for breast cancer while consumption of choline plus betaine could reduce the risk of cancer incidence by 58%. Such results may be due to not enough studies on the relationship between dietary choline and betaine consumption and cancer risk to be summarized to obtain more reliable results. Different results also appeared when stratified by publication year and quality score of the study. The former could be explained by differences from qualities of scientific researches in different eras, and as to the latter, the relationship may have been attenuated by poor methodological quality of studies.

To our knowledge, meta-analysis is a crucial method for revealing trends that might not be evident in a single study. One of strengths of this meta-analysis is that the number of total subjects (513,390) was substantial, which significantly increased the statistical power. Moreover, a significant dose-response relation was observed between choline plus betaine intake and cancer risk, further strengthening the relationship. We also conducted sensitivity analyses and the combined estimates were robust except the one for choline plus betaine consumption and cancer risk. The reason for this phenomenon may be interpreted by the very inconsistent ranges of exposure in the studies by Zeng *et al.*^9^, Zhang *et al.*^10^, Lu *et al.*^11^ and Kotsopoulos *et al.*^13^.

Several limitations exist in our meta-analysis, though. First, in observational studies, the possibility that residual confounders may account for the protective effect of choline and betaine could not be ruled out. Nevertheless, all studies except the one by Xu *et al.*^6^ included in this meta-analysis have adjusted for a wide spectrum of potential confounders, like age, gender, total energy intake, folate consumption, smoking, alcohol drinking and so on. Second, the distribution of the highest and lowest choline and betaine consumption is not uniform among studies, which may conduce to the heterogeneity in the pooled analysis and conclusions limited. Third, this present meta-analysis contains a small number of observational studies and only 2 case-control studies containing 3 comparisons did not fail to meet the applied conditions of the dose-response meta-analysis. Therefore, more observational studies with standardized choline and betaine consumption collection strategies are needed to answer the two questions more completely. Fourth, a problem with dietary assessments in case-control studies is that dietary patterns and habits may have changed after the diagnosis of cancer because of symptoms related to the cancer. Fifth, many studies only provided the results without showing detailed calculation methods or the raw data (e.g., the number of case and non-case or person-year at different ranges of consumption) and we contacted the corresponding authors for the data, but unfortunately, we failed to get any reply. So the investigators of all the published studies are encouraged to share their raw data. Sixth, substantial heterogeneity was explored among the comparisons in our meta-analysis, which could be mostly explained by study design, location and publication year according to the meta-regression analysis. Finally, publication bias appeared when examining the relationship between consumption of choline only and cancer risk and the tendency of small studies with null results not being published could be the fairly reasonable explanation.

Cancer is a large burden worldwide. The relationship between dietary choline and betaine consumption and the risk of cancer remains controversial. The results of this meta-analysis indicate that a 100 mg/d increment in consumption of choline plus betaine decreases cancer occurrence by 11%, which means that the above mentioned approximately 14.1 million new cancer cases in 2012 worldwide could fall to about 12.5 million. Besides, choline must be consumed through the diet to play its role of maintaining health of humans, though choline can also be synthetised by the liver^29^. Dietary sources of choline are mainly eggs, beef, pork, liver, soybean, and wheat germ^30^, while we can obtain betaine mainly from wheat bran, wheat germ, and spinach^31^. However, if the recommended dietary adequate intake (550 mg/day for men and 425 mg/day for women) for choline^32^ is took as a standard, there are only a minority of people meeting it. Some studies have reported that 20–25% of Americans are not up to that ‘standard’. For exmaple, in the Framingham Heart Study^33^, the Atherosclerosis Risk In Communities study^34^,^35^ and the Nurse’s Health Study^36^. the amount of dietary choline consumption is <203 mg/d, < 217 mg/d and <293 mg/d, respectively. Choline and betaine intake may be increased by well-described dietary changes such as appropriate increases in consumption of the aforementioned foods.

To conclude, our meta-analysis manifests that choline and betaine consumption possesses the ability to lower cancer risk. However, these results should be considered with caution on account of the considerable heterogeneity, the potential biases and confounding factors. Further studies well designed as large prospective studies and placebo-controlled intervention trials on choline and betaine supplementation are warranted to ascertain the results and establish the potential dose-response relationship. For better and comprehensive understanding of the relationship between dietary consumption of choline and betaine and cancer risk, future studies are expected to use standardized collection strategy for choline and betaine consumption. Furthermore, it also requires further studies to elucidate the underlying mechanisms responsible for the relationship.

## Methods

### Data sources and searches

We retrieved epidemiologic studies examining the relationship between dietary choline and betaine intake and cancer risk from two databases (PubMed and EMBASE), from their inception to March 2016, using the following key words: (dietary OR consumption OR intake OR food) AND (choline OR betaine) AND (cancer OR tumor OR carcinoma OR neoplasm). The search was limited to no language restriction. In addition, the references of identified publications were scrutinised for further potentially relevant articles. This present meta-analysis was designed, performed and reported on the basis of the epidemiology guidelines for meta-analysis of observational studies^37^.

### Study selection criteria

Published articles were included if they were in accordance with the inclusive criteria: 1) the study design was case-control or cohort; 2) the exposure of interest was dietary consumption of choline or betaine or choline plus betaine; 3) the outcome was cancer occurrence; 4) reported the relative risk (RR) or odds ratio (OR) with its 95%CI for the relationship between the consumption of choline or betaine or choline plus betaine and cancer risk adjusted at least for age. In case that more than one published paper reported data based on the same population, we selected the most recent or most informative one. Two of the authors independently identified and reviewed each relevant article, and discrepancies in study eligibility were discussed until an agreement of opinions was reached.

### Data extraction and quality assessment

The relevant information was extracted by 2 of the authors independently. The extracted information for each study was as follows: the last name of the first author, year of publication, type of cancer, design of the study, the location of the study conducted in, age, gender, sample sizes, the assessment and comparison method for the consumption of choline and betaine, RRs or ORs and the corresponding 95% CIs (the highest versus the lowest consumption of choline only or betaine only or choline plus betaine) from the most fully adjusted model and adjustment for confounders in a multivariate analysis.

In order to evaluate the quality of the study, a 9-star system based on the Newcastle-Ottawa Scale^38^ which contains 3 aspects (the selection of study populations, the comparability of the populations and ascertainment of exposure or the outcomes of interest for case-control or cohort studies) was adopted to judge on the study. Additionally, if a study with data analysis using the residual method^39^ could achieve an additional star. In other words, the maximum score was 10 stars and when a study was assessed with ≥7 stars, it would be regarded as a high-quality study.

### Statistical analysis

Adopting random effect models, which takes into account both within-study and between-study variations^40^, we estimated the pooled RRs with 95% CIs by summarizing the risk estimates of each study. Heterogeneity among studies was evaluated using the Cochran’s Q test and *I^2^* (inconsistency index) statistic^41^. A value of I^2^ greater than 50% indicates severe heterogeneity and the value less than 25% suggesting no significant heterogeneity^42^. Based on the location, study design, cancer type, publication year, sex and quality score of study, we carried out stratified analyses to explore possible heterogeneity and test the robustness of the relationship. In the meta-regression model, we examined the variables of study design, location, and publication year to explore the possible heterogeneity. Furthermore, by omitting one comparison at every turn, we also undertook sensitivity analyses to assess the effect of a single comparison over the overall risk estimates.

For the dose-response analysis, the method of generalised least square for trend estimation put forward by Greenland and Longnecker^43^ and Orsini *et al.*^44^ was adopted to estimate the trend from the correlated log RR estimates across categories of choline plus betaine intake. It is necessary for the method that the studies provided data of distribution of cases and non-cases or person-years and the RR or OR with its 95% CI known for at least 3 quantitative exposure categories as well as the median or mean values of choline plus betaine in each category. When studies merely provided the consumption data by ranges, we considered the average of the lower and upper bound as estimation of the midpoint of each category. Supposing that the two ends of the categories were both open ended, we regarded the length of the open interval to be the same as that of the closest interval for the highest category and set the lower boundary to zero for the lowest.

We also adopted Begg’s funnel plots and Egger’s linear regression test^45^,^46^ to evaluate whether publication bias existed. All the statistical analyses involved were performed with STATA software (version 13.1; StataCorp, College Station, Texas, USA). *P* values were two-sided and *P* < 0.05 was considered statistically significant.

## Additional Information

**How to cite this article**: Sun, S. *et al.* Choline and betaine consumption lowers cancer risk: a meta-analysis of epidemiologic studies. *Sci. Rep.* **6**, 35547; doi: 10.1038/srep35547 (2016).

## Supplementary Material

Supplementary Information

## Figures and Tables

**Table 1:** Characteristics of studies on choline and betaine consumption and cancer risk.

First author year of publication (reference)	Cancer type	Country/design	Cases/Controls	Age(y)/sex	Follow-up (y)	Assessment of consumption/food item number/choline and betaine consumption calculation/nutrient database	Contrast (highest vs lowest)			Ajusted OR/RR(95%CI) (highest vs lowest)			Matched or adjusted variables
							Choline consumption only	Betaine consumption only	Choline plus Betaine consumption	Choline consumption only	Betaine consumtion only	Choline plus Betiane consumption	
Xu *et al.*^6^	Breast cancer	USA; case-control (PB)	1,508/1,556	20-98 F	NR	FFQ/100/frequency × portion-size/USDA database	Quintile >247.2 vs < 122.7mg/d	Quintile >179.71vs < 61.11mg/d	NR	0.85(0.61–1.18)	0.96(0.73–1.25)	NR	Age and daily energy intake
Ibiebele *et al.* 2010^7^	Esophageal Cancer	Australia; case-control (PB)	881/1,507	18-79 M/F	NR	FFQ/135/-/USDA database	Quartile 1171 vs 380mg/d	Quartile 185 vs 85mg/d	NR	EAC: 1.25(0.91–1.72) ESCC: 1.39(0.88–2.30)	EAC: 0.79(0.58–1.07) ESCC: 1.30(0.83–1.99)	NR	Age, gender, education, BMI 1 y previously, frequency of heartburn or acid reflux 10 y prior to diagnosis, lifetime alcohol intake, pack-years of smoking, NSAID use, and total energy intake
Ying *et al.*^8^	Lung Cancer	USA; case-control (HB)	2,821/2,923	no age restrictions M/F	NR	FFQ/214/frequency × nutrient content/USDA database	Quartile (highest vs lowest)	Quartile (highest vs lowest)	NR	0.92(0.78–1.07)	0.71(0.61–0.84)	NR	Sex, race/ethnicity, age, pack-years, total caloric intake, family cancer history, dust exposure, second-hand smoke, emphysema, hay fever, smoking status, asthma, addiction index, alcohol, time since smoking cessation
Zeng *et al.*^9^	Nasopharyngeal Carcinoma	China; case-control (HB)	600/600	30–75 M/F	NR	FFQ/78/frequency × portion-size/Chinese Food Composition Table	Quartile 285 vs 125mg/d	Quartile 539 vs 112mg/d	Quartile 761 vs 285mg/d	0.42(0.29–0.61)	0.50(0.35–0.72)	0.44(0.30–0.64)	Age, BMI, occupation, marital status, educational level, household income, current smoking, current drinking, exposure to potential toxic substances, multivitamin supplement, chronic rhinitis history, physical activity, dietary folate intake and daily energy intake
Zhang *et al.*^10^	Breast cancer	China; two stage case-control (HB)	stage 1: 438/438 stage 2: 369/369 Pooled: 807/807	25–70 F	NR	FFQ/81/frequency × portion-size/Chinese Food Composition Table	stage 1: Quartile >207.86 vs < 111.64mg/d stage 2: Quartile >232.24 vs < 123.66mg/d Pooled: Quartile >217.47 vs < 117.65mg/d	stage 1: Quartile >440.90 vs < 279.40 mg/d stage 2: Quartile >385.28 vs < 154.75 mg/d Pooled: Quartile >408.04 vs < 140.14 mg/d	stage 1: Quartile >627.38 vs < 279.40 mg/d stage 2: Quartile >600.19 vs < 314.00 mg/d Pooled: Quartile >615.63 vs < 297.57 mg/d	stage 1: 0.35(0.22–0.54) stage 2: 0.51(0.30–0.87) Pooled: 0.40(0.28–0.57)	stage 1: 0.60(0.39–0.92) stage 2: 0.45(0.25–0.80) Pooled: 0.58(0.42–0.80)	stage 1: 0.43(0.28–0.66) stage 2: 0.40(0.22–0.71) Pooled: 0.38(0.27–0.53)	Occupation, body mass index, age at menarche, live births and age at first live birth, mother/sister/daughter with breast cancer, passive smoking, alcohol consumption, physical activity, total energy intake and study stage
Lu *et al.*^11^	Colorectal Cancer	China; case-control (MB)	890/890	30–75 M/F	NR	FFQ/81/frequency × portion-size/Chinese Food Composition Table	Quartile >176.40 vs < 99.68mg/d	Quartile >323.10 vs < 116.70mg/d	Quartile >535.50 vs < 224.70mg/d	0.54(0.37–0.80)	0.92(0.65–1.29)	0.91(0.65–1.28)	Age, sex, residence, marital status, education, income level, occupation, family history of cancer, smoking status, passive smoking, alcohol drinking, degree of physical activity, BMI, red meat, fish, beans and folate intake
Cho *et al.*^12^	Breast cancer	USA; cohort	1,032/89,631	26–46 F	12	FFQ/about 130/-/USDA database or other sources	Quintile 397 vs 267mg/d	Quintile 305 vs 114mg/d	NR	0.88(0.72–1.07)	0.99(0.79–1.22)	NR	Smoking, height, parity and age at first birth, body mass index, age at menarche, family history of breast cancer, history of benign breast disease, oral contraceptive use, and intakes of alcohol, energy, and animal fat
Kotsopoulos *et al.*^13^	Epithelial ovarian cancer	USA; cohort	526/159,431	25–55 F	22	FFQ/-/-/USDA database and other sources	Quintile NHS:≥ 338.8 vs < 249.5 mg/d NHSII: ≥367.3 vs < 269.7 mg/d	Quintile NHS: ≥127.3 vs < 70.5 mg/d NHSII: ≥138.9 vs >80.6 mg/d	Quintile NHS: ≥453.8 vs < 339.8 mg/d NHSII: ≥491.7 vs < 371.6 mg/d	0.98(0.73–1.31)	0.98(0.74–1.31)	0.99(0.75–1.32)	Age at menarche and parity, duration of oral contraceptive use, tubal ligation, height, family history of breast or ovarian cancer, caffeine intake, hysterectomy, PMH/menopausal status, caloric intake, alcohol consumption and BMI
Lee *et al.*^14^	Colorectal Cancer	USA; cohort	987/46,315	40–75 M	18	FFQ/-/frequency × nutrient content/USDA database	Quintile (highest vs lowest)	Quintile (highest vs lowest)	NR	0.97(0.79–1.20)	0.94(0.77–1.16)	NR	Total energy intake, aspirin dose, pack-years of smoking before age 30, body mass index, family history of colorectal cancer, history of endoscopy, alcohol intake, and total folate
Cho *et al.*^15^	Breast cancer	USA; cohort	3,990/70,594	30–55 F	20	FFQ/about 130/frequency × nutrient content/Harvard University Food Composition Database and USDA database	Quintile 396 vs 260mg/d	Quintile 144 vs 71mg/d	NR	1.10(0.99–1.22)	0.98(0.89–1.09)	NR	Smoking status, height, parity and age at first birth,body mass index at age 18, weight change between age 18 and current, physical activity, age at menarche, family history of breast cancer, history of benign breast disease, use of post-menopausal hormones, and intakes of alcohol, energy, and folate
Cho *et al.*^16^	Renal cell cancer	USA; cohort	NHS: 225/76,983 HPFS: 221/47,665	NHS: 30–55 F HPFS: 40–75 M	F: 24 M: 22	FFQ/about 131/frequency × nutrient content/USDA database and other sources	NHS: Quintile 399.8 vs 267.1mg/d HPFS: Quintile 471.7 vs 308.3mg/d Pooled: Quintile (highest vs lowest)	NHS: Quintile 138.4 vs 70.5mg/d HPFS: Quintile 186.4 vs 84.9mg/d Pooled: Quintile (highest vs lowest)	NR	NHS: 0.78(0.48–1.27) HPFS: 0.96(0.62–1.48) Pooled: 0.87(0.63–1.21)	NHS: 0.78(0.49–1.24) HPFS: 1.30(0.83–2.02) Pooled: 1.01(0.62–1.65)	NR	Age, smoking status, body mass index, history of hypertension, history of diabetes, physical activity, fruit intake, vegetable intake, and alcohol intake in NHS and HPFS and parity in NHS

Abbreviations: EAC, esophageal adenocarcinoma; ESCC, esophageal squamous cell carcinoma; FFQ, food frequency questionnaire; HB, hospital-based; HPFS, The Health Professionals Follow-up Study; PB, population-based; MB, mixed based (including both hospital-based and population-based); NASID, Non-steroidal Anti-inflammatory Drugs; NHS, The Nurses’ Health Study; NHSII, The Nurses’ Health Study II; NR, no record; PMH, postmenopausal hormone; USDA, US Department of Agriculture.

**Table 2:** Stratified analyses of dietary choline and betaine consumption and cancer risk.

Group	Choline consumption only					Betaine consumption only					Choline plus betaine consumption				
	No. of comparisons	Summary OR(95%CI)	Heterogeneity			No. of comparisons	Summary OR(95%CI)	Heterogeneity			No. of comparisons	Summary OR(95%CI)	Heterogeneity		
			***χ^2^***	***P***	***I^2^(%)***			***χ^2^***	***P***	***I^2^(%)***			***χ^2^***	***P***	***I^2^(%)***
Total	14	0.82(0.69–0.96)	65.56	< 0.001	80.2	14	0.86(0.76–0.97)	38.02	<0.001	65.8	5	0.60(0.40–0.90)	22.23	<0.001	82.0
**Design**															
Cohort	6	1.00(0.92–1.10)	5.68	0.339	11.9	6	0.98(0.90–1.06)	2.64	0.755	0	1	0.99(0.75–1.31)	0	—	—
Case-control	8	0.71(0.52–0.97)	47.07	<0.001	85.1	8	0.75(0.62–0.92)	20.09	0.005	65.1	4	0.52(0.34–0.80)	12.17	0.007	75.3
Population based	3	1.11(0.83–1.49)	3.88	0.144	48.4	3	0.96(0.75-1.23)	—	0.185	40.7	0	—	—	—	—
Hospital based	4	0.52(0.30–0.90)	28.75	<0.001	89.6	4	0.60(0.49–0.75)	4.87	0.181	38.4	3	0.43(0.33–0.55)	0.07	0.965	0
Mixed based	1	0.54(0.37–0.79)	0	—	—	1	0.92(0.65–1.30)	0	—	—	1	0.91(0.65–1.28)	0	—	—
**Location**															
Non-China	10	0.99(0.91–1.08)	11.55	0.24	22.1	10	0.93(0.83–1.03)	18.49	0.030	51.3	1	0.99(0.75–1.31)	0	—	—
China	4	0.45(0.36–0.55)	2.41	0.492	0	4	0.61(0.44–0.85)	7.60	0.055	60.5	4	0.52(0.34–0.80)	12.17	0.007	75.3
**Cancer type**															
Breast cancer	5	0.73(0.52–1.01)	32.51	<0.001	87.7	5	0.86(0.71–1.04)	11.24	0.024	64.4	2	0.42(0.30–0.59)	0.04	0.845	0
Other	9	0.86(0.70–1.06)	31.42	<0.001	74.5	9	0.86(0.73–1.02)	22.43	0.004	64.3	3	0.74(0.46–1.20)	12.29	0.002	83.7
**Publication year**															
≤2010	7	1.02(0.92–1.14)	8.54	0.201	29.8	7	0.97(0.90–1.04)	3.62	0.727	0	1	0.99(0.75–1.31)	0	—	—
>2010	7	0.61(0.44–0.84)	32.7	<0.001	81.7	7	0.72(0.57–0.90)	16.02	0.014s	62.5	4	0.52(0.34–0.80)	12.17	0.007	75.3
**Sex**															
M only	2	0.97(0.80–1.17)	0	0.966	0	2	1.04(0.77–1.39)	1.68	0.194	65.8	0	—	—	—	—
F only	7	0.78(0.61–0.99)	33.29	<0.001	82.0	7	0.88(0.76–1.02)	11.96	0.063	49.8	3	0.57(0.30–1.09)	14.24	0.001	86.0
M and F	5	0.81(0.55–1.20)	29.71	<0.001	86.5	5	0.78(0.62–1.00)	12.86	0.012	68.9	2	0.64(0.31–1.30)	7.85	0.005	87.3
**Quality score**															
<7 stars	4	1.03(0.84–1.26)	5.61	0.132	46.5	4	0.87(0.69–1.10)	8.64	0.034	65.3	0	—	—	—	—
≥7 stars	10	0.73(0.58–0.91)	59.27	<0.001	84.8	10	0.85(0.73–0.99)	25.55	0.002	64.8	5	0.60(0.40–0.90)	22.23	<0.001	82.0
