# Supplementary material for: Choline and betaine consumption lowers cancer risk: a meta-analysis of epidemiologic studies
Source: Sci Rep. 2016 Oct 19;6:35547. doi: 10.1038/srep35547 (PMC5069558; doi:10.1038/srep35547)
Supplement: Supplementary Information [file srep35547-s1.doc]

**Choline and betaine consumption lowers cancer risk : a meta-analysis of epidemiologic studies**

Shanwen Sun^1#^, Xiao Li^2#^ ,Anjing Ren^1#^, Mulong Du^3,4^, Haina Du^5^, Yongqian Shu^1^, Lingjun Zhu^1^*, Wei Wang^6^*

^1^Department of Oncology, The First Affiliated Hospital of Nanjing Medical University, Nanjing, China; ^2^Department of Pathology, The First Affiliated Hospital of Nanjing Medical University, Nanjing, China; ^3^Department of Environmental Genomics, Jiangsu Key Laboratory of Cancer Biomarkers, Prevention and Treatment, Cancer Center, Nanjing Medical University, Nanjing, China; ^4^Department of Genetic Toxicology, the Key Laboratory of Modern Toxicology of Ministry of Education, School of Public Health, Nanjing Medical University, Nanjing, China; ^5^Department of Oncology, Nanjing Hospital of T.C.M, Nanjing, China; ^6^The First Affiliated Hospital of Nanjing Medical University, Nanjing, China.

^#^ These authors contributed equally to this work.

*Corresponding author:

Lingjun Zhu: Department of Oncology, The First Affiliated Hospital of Nanjing Medical University, 300 GuangZhou Rd, Nanjing 210029, Nanjing, China; Cell phone: +13951807457; E-mail address: zhulingjun@njmu.edu.cn;

Wei Wang: Department of Thoracic Surgery, The First Affiliated Hospital of Nanjing Medical University, 300 GuangZhou Rd, Nanjing 210029, Nanjing, China; Cell phone: +15261883958; E-mail address: wangwei-doctor@163.com.

**Supplemental Figure 1. Sensitivity analysis of the pooled relative risk coefficients on the relationship between choline consumption and cancer risk.** EAC, esophageal adenocarcinoma; ESCC, esophageal squamous cell carcinoma; I, stage one; II stage two; HPFS, The Health Professionals Follow-up Study; NHS, The Nurses’ Health Study; Bars, 95% CI.

**Supplemental Figure 2.** **Sensitivity analysis of the pooled relative risk coefficients on the relationship between choline consumption and cancer risk.** EAC, esophageal adenocarcinoma; ESCC, esophageal squamous cell carcinoma; I, stage one; II stage two; HPFS, The Health Professionals Follow-up Study; NHS, The Nurses’ Health Study; Bars, 95% CI.

**Supplemental Figure 3.** **Sensitivity analysis of the pooled relative risk coefficients on the relationship between choline consumption and cancer risk.** I, stage one; II stage two; Bars, 95% CI.

**Supplemental Table 1. Methodological quality of cohort studies included in the meta-analysis¹.**

| First author year of publication (reference) | Selection | | | | Comparability | Exposure | | | Data analysis that used  a residual method | Total quality   scores |
| --- | --- | --- | --- | --- | --- | --- | --- | --- | --- | --- |
|  | Adequate definition  of the cases | Representativeness  of the cases | Selection of Controls | Definition of  Controls | Control for important factor² | Ascertainment  of exposure | Same method of ascertainment  for cases and controls | Non-Response  rate³ |  |  |
|  |  |  |  |  |  |  |  |  |  |  |
| Xu et al. 2009^6^ | ☆ | ☆ | ☆ | ☆ | ☆ | - | ☆ | - | - | 6 |
| Ibiebele et al. 2010^7^ | ☆ | - | ☆ | ☆ | ☆ | - | ☆ | - | ☆ | 6 |
| Ying et al. 2013 ^8^ | ☆ | ☆ | - | ☆ | ☆ | - | ☆ | - | ☆ | 6 |
| Zeng et al. 2013^9^ | ☆ | ☆ | - | ☆ | ☆☆ | - | ☆ | - | ☆ | 7 |
| Zhang et al. 2013^10^ | ☆ | ☆ | - | ☆ | ☆☆ | - | ☆ | - | ☆ | 7 |
| Lu et al. 2015^11^ | ☆ | ☆ | - | ☆ | ☆☆ | - | ☆ | ☆ | ☆ | 8 |

**¹** A study could be awarded a maximum of one star for each item except for the item Control for important factor or additional factor.

**²**A maximum of 2 stars could be awarded for this item. Studies that controlled for age received one star, whereas studies that controlled for consumption of other nutrients such as folate received an additional star.

**³** One star was assigned if there was no signiﬁcant difference in the response rate between control subjects and cases by using the chi-square test (P＞0.05).

**Supplemental Table 2. Methodological quality of case-control studies included in the meta-analysis¹.**

| First author year of publication (reference) | Selection | | | | Comparability | Outcome | | | Data analysis that used  a residual method | Total quality  scores |
| --- | --- | --- | --- | --- | --- | --- | --- | --- | --- | --- |
|  | Representativeness of the exposed cohort | Selection of the unexposed cohort | Ascertainment of exposure | Outcome of interest not present  at start of study | Control for important factor² | Assessment of outcome | Follow-up long enough for outcomes to occur³ | Adequacy of follow-up  of cohorts⁴ |  |  |
|  |  |  |  |  |  |  |  |  |  |  |
| Cho et al. 2007^12^ | ☆ | ☆ | ☆ | ☆ | ☆☆ | ☆ | ☆ | - | ☆ | 9 |
| Kotsopoulos et al. 2009^13^ | ☆ | ☆ | ☆ | ☆ | ☆☆ | ☆ | ☆ | - | ☆ | 9 |
| Lee et al. 2010^14^ | ☆ | ☆ | ☆ | ☆ | ☆☆ | ☆ | ☆ | - | ☆ | 9 |
| Cho et al. 2010^15^ | ☆ | ☆ | ☆ | - | ☆☆ | ☆ | ☆ | ☆ | ☆ | 9 |
| Cho et al. 2013^16^ | ☆ | ☆ | ☆ | ☆ | ☆ | ☆ | ☆ | ☆ | ☆ | 9 |

**¹** A study could be awarded a maximum of one star for each item except for the item Control for important factor or additional factor.

**²**A maximum of 2 stars could be awarded for this item. Studies that controlled for age received one star, whereas studies that controlled for consumption of other nutrients such as folate received an additional star.

³ A cohort study with a follow-up time>10y was assigned one star.

⁴ A cohort study with a follow-up rate>80% was assigned one star.

**Supplemental Table 3. Meta-regression analysis.**

|  | Choline consumption only | | | | Betaine consumption only | | | |
| --- | --- | --- | --- | --- | --- | --- | --- | --- |
| Variable | Coefficient | Standard error | *P* value | 95% CI | Coefficient | Standard error | *P* value | 95% CI |
| Study design | 0.29 | 0.204 | 0.181 | -0.155 to 0.734 | 0.253 | 0.113 | **0.045** | 0.007 to 0.500 |
| Location | 0.797 | 0.121 | **<** **0.001** | 0.533 to 1.06 | 0.398 | 0.142 | **0.016** | 0.088 to 0.708 |
| Publication year | 0.505 | 0.158 | **0.008** | 0.162 to 0.849 | 0.302 | 0.09 | **0.006** | 0.106 to 0.498 |

NOTE. Values in bold are statistically significant in meta-regression analysis.
